# Exploiting Pre-Trained Convolutional Neural Networks for the Detection of Nutrient Deficiencies in Hydroponic Basil

**DOI:** 10.3390/s23125407

**Published:** 2023-06-07

**Authors:** Zeki Gul, Sebnem Bora

**Affiliations:** Department of Computer Engineering, Ege University, 35100 Izmir, Turkey; gulzeki96@gmail.com

**Keywords:** basil plant, convolutional neural network, hydroponic cultivation, nutrient deficiencies, transfer learning

## Abstract

Due to the integration of artificial intelligence with sensors and devices utilized by Internet of Things technology, the interest in automation systems has increased. One of the common features of both agriculture and artificial intelligence is recommendation systems that increase yield by identifying nutrient deficiencies in plants, consuming resources correctly, reducing damage to the environment and preventing economic losses. The biggest shortcomings in these studies are the scarcity of data and the lack of diversity. This experiment aimed to identify nutrient deficiencies in basil plants cultivated in a hydroponic system. Basil plants were grown by applying a complete nutrient solution as control and non-added nitrogen (N), phosphorous (P) and potassium (K). Then, photos were taken to determine N, P and K deficiencies in basil and control plants. After a new dataset was created for the basil plant, pretrained convolutional neural network (CNN) models were used for the classification problem. DenseNet201, ResNet101V2, MobileNet and VGG16 pretrained models were used to classify N, P and K deficiencies; then, accuracy values were examined. Additionally, heat maps of images that were obtained using the Grad-CAM were analyzed in the study. The highest accuracy was achieved with the VGG16 model, and it was observed in the heat map that VGG16 focuses on the symptoms.

## 1. Introduction

Developments in the fields of artificial intelligence and communication have enabled the new industrial revolution called Industry4.0 to improve production using systems that can communicate with each other and have the ability to detect and intervene in the environment. This development in modern industry has affected many areas (education, health, security) and agricultural production. As a result, autonomous or non-autonomous methods such as geographical information systems, early warning and recommendation systems, and monitoring and control systems to support production in agriculture have started to be applied under the name Agriculture 4.0 [1].

The proper use of natural resources such as water and soil in agricultural production has been an important issue over the last 20 years, after being a subject of research in the past, with warnings about diminishing resources and environmental damage. Several attempts have been made to increase yields in production via methods such as mechanization, chemical use and fertilization. However, these implementations have caused environmental, social and economic problems over time. The amount of arable land is decreasing due to the improper use of fertilizers and chemicals, improper crop rotation and negligent use of natural resources. In addition, climate change, considered both cause and effect, has reduced the yield and quality of agricultural products [2]. Meanwhile, studies have been carried out to develop different kinds of agricultural production techniques; to use fertilizers according to the result of the analysis of the elements in plants, soil and water; to perform irrigation according to the plant’s need for water [3,4]; to watch the weather conditions [5,6]; to detect plant pests (insects, weeds, etc.) [7,8] and to detect damage that has already occurred [9,10]. 

Soilless culture, one of the production techniques in agriculture, is based on the principle of fulfilling the soil’s function in plant growth as the natural growing environment for plants. The nutrient and water needs of plants are provided via nutrient solution application. In regions where environmental conditions such as the climate, water and soil are unfavorable for agriculture, soilless cultivation techniques can be used to grow products of high yield and quality. The controlled nutrition of plants in soilless culture and the reuse of nutrient solutions in some soilless culture techniques ensure the proper use of resources [11]. The controlled environment created for soilless agriculture reduces the amount of the parameters that need to be controlled and followed. This makes soilless cultivation suitable for being equipped with smart technologies that can sense and respond to environmental conditions. 

Some nutrients need to be fully supplied to the plant in order to grow with high yield and quality. These nutrients are carbon (C), hydrogen (H), oxygen (O), nitrogen (N), phosphorus (P), potassium (K), calcium (Ca), magnesium (Mg), sulfur (S), chlorine (Cl), copper (Cu), iron (Fe), manganese (Mn), molybdenum (Mo), zinc (Zn) and boron (B). The plant, which has an absolute need for each element given above, presents various symptoms in the absence of these nutrients, giving growers and researchers an idea about the necessary nutritional supplement (fertilizer). With nitrogen deficiency (N-), older leaves have a lighter color than the other parts of the plant. With phosphorus deficiency (P-), the development of plants and fruits decreases, and brownness between the veins is observed in old leaves, while underdevelopment and a dark green color are observed in young leaves. With potassium deficiency (K-), the tips of plant leaves turn yellow and curl, and in plants with fruits, the fruits are deformed and discolored. With calcium deficiency (Ca-), young leaves turn yellow and leaf margins curl, while with magnesium deficiency (Mg-), discoloration between leaf veins is observed [12]. Since the plant shows visible symptoms in deficiencies of other elements as well as these elements, growers/researchers can guess which element is deficient. As visual diagnosis can be used to identify nutrient deficiencies, a system capable of analyzing the plant images or symptom images will help prevent economic losses due to nutritional deficiencies.

The fact that plants show physical symptoms of nutritional deficiencies has encouraged artificial intelligence (AI) researchers to work on this topic. Many studies have been published in the literature to show that nutrient deficiencies in plants can be predicted, and necessary recommendations on fertilization can be made. In addition, the high performance of deep learning applications in image processing with convolutional neural networks (CNNs) has inspired many researchers to exploit CNNs for the detection of plant disorders [13]. For the detection of healthy plants and nitrogen, phosphorus and potassium deficiencies in plants, Yi [14] carried out studies with sugar beet, Wulandhari [15] with okra, Guerrero [16] with banana, Sharma [17] with rice and Taha [18] with lettuce grown in water culture. Researchers that used pre-trained neural networks, i.e., models called Transfer Learning (DenseNet, NasNet, InceptionResnet, VGG, and GoogleNet), were able to make predictions that demonstrated an accuracy above 86%. Kusanur [19] detected magnesium and calcium deficiency in tomato plants, while Rahadiyan [20] detected potassium, calcium, magnesium and sulfur deficiency in chili pepper. Due to the variety and lack of data in the studies, processes such as resizing, shearing, rotation, scaling, mirroring and cropping in images were observed to improve the results, while processes such as changing the color scale or adding noise reduced the accuracy [14]. Apart from these processes, it was shown by many researchers, including Taha [18], Islam [21], Yang [22], Ngugi [23] and Azimi [24], that the performance of CNN models increased when the background of images was removed. 

Azimi [24] determined the level of nitrogen deficiency in sorghum plants, where nitrogen deficiency at different rates (100%, 50% and 10%) was applied. It consists of different color scales, such as RGB and NIR, in a dataset consisting of 96,867 photographs. In this study, machine learning models Resnet18, Nasnet and their designed deep learning models were compared. They obtained an accuracy value of 0.84 with the model they designed, which gave a better result than machine learning models and was close to pre-trained deep learning models. Additionally, the study showed that background cleaning improves the result. 

Sharma [17] detected nitrogen, phosphorus and potassium deficiencies in the rice plant with the model he built from combinations of different pre-trained CNNs. In this study, which started with 11 ready-made models, 2 and 3 combinations of 4 models (InceptionResNetV2, VGG19, DenseNet201 and Xception) and all of them were compared. The best accuracy result was obtained from the combination of InceptionResNetV2 and DenseNet201 with 96%.

In Taha’s study [18] which saw images taken from lettuce grown in a hydroponic system, 96% accuracy was obtained with Inceptionv3. The study, in which nitrogen, phosphorus and potassium deficiencies were detected, sets an example in terms of the applicability of autonomous systems.

Since we found no study on basil plants after our research, we set up an experiment based on the deficiencies of nitrogen, phosphorus, potassium, calcium and magnesium elements in basil. Within the scope of the study, a dataset was created for healthy plants and plants with nitrogen, phosphorus and potassium deficiencies, and then the transfer-learning-based CNN models capable of making predictions for these four classes were compared for their success in classification and their performance in feature detection. Section 2 of this paper covers the creation of the dataset, basic information about the methods used and the training of the models; Section 3 covers the evaluation of the training, testing and Grad-CAM results and Section 4 covers the paper’s conclusion and future studies.

## 2. Materials and Methods

**Figure 1:**
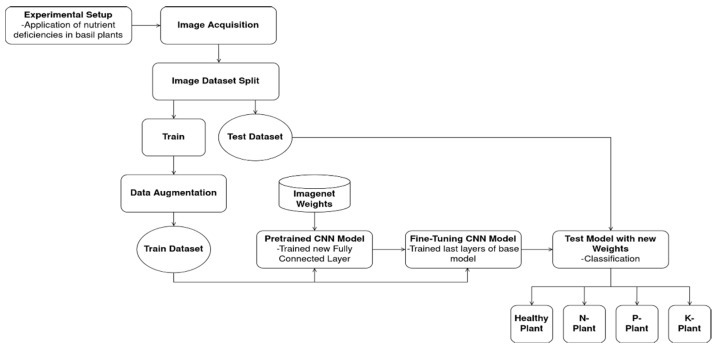
Methodology of the study.

The phases of study (Figure 1) consisted of setting up the experiment with nutritional deficiencies in basil plant; collecting photographs of symptomatic plants; creating the dataset; designing the fully connected layer; training the models; applying fine-tuning and testing the models.

### 2.1. Experimental Setup

**Figure 2:**
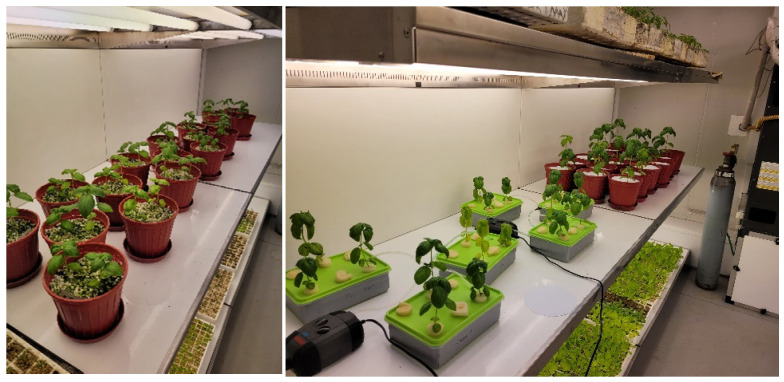
Substrate culture (**left**) and hydroponic (**right**).

A controlled environment is required for the application of nutrient deficiencies. Therefore, the experiment (Figure 2) was set up with 36 seedlings by planting 2 seedlings in 3 pots in a perlite medium to observe macro element deficiencies using soilless agriculture techniques.

At the end of the second week, 1 plant from each pot was transferred to a hydroponic medium (water culture), and 3 plants were grown in a perlite medium (substrate culture) and 3 plants were grown in a hydroponic medium for 4 weeks.

In hydroponic culture, the solution in the container was changed once a week. The nutrient solution was continuously aerated using an aquarium pump. In substrate culture, plants were watered with 100 mL of the solution every 4 days. The nutrient solution prepared according to the recommended recipe (Table 1) for vegetables with edible leaves as well as aromatic plants was taken as a reference. Distilled water was used for nutrient solution preparation.

The solution described in Table 1 was used in all plants for one week after planting. At the end of one week, lacking nutrient solutions were prepared via macro element (N, P, K, Ca and Mg) deficiencies, which constitutes the subject of the experiment. Following that, the implementation of nutrient deficiencies started with these 6 nutrient solutions. 

**Figure 3:**
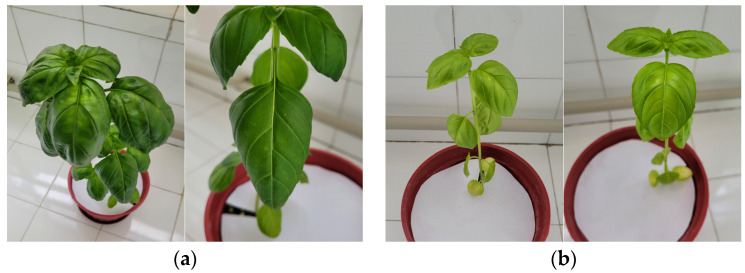

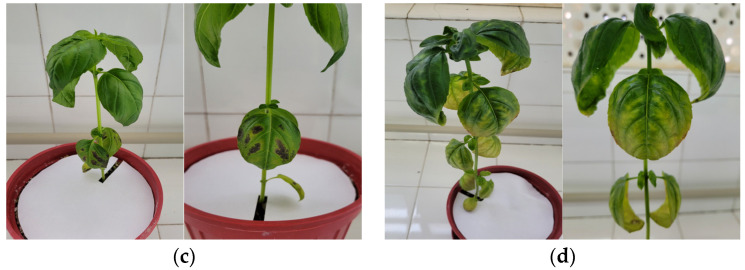
Plant images in dataset; (**a**) healthy (control) plant, (**b**) N deficiency (N-), (**c**) P deficiency (P) and (**d**) K deficiency (K-).

A healthy plant (Figure 3a) has green leaves and a developed stem. In healthy plants, more stem and leaf developments are observed compared to other experiments. 

In the case of N- (Figure 3b), there is chlorosis (yellowing) of the whole plant. Moreover, the plant is underdeveloped compared to a healthy plant. 

In the case of P- (Figure 3c), symptoms usually develop, with purple/black spots on older/lower leaves. There is no deformity in younger/upper leaves.

K- (Figure 3d) developed in older/lower leaves of the plant with an increase in yellow and brown shades similar to sunburn, first in spots and then on the leaf margins and between the veins on the leaf.

### 2.2. Dataset

#### 2.2.1. Image Acquisition

**Figure 4:**
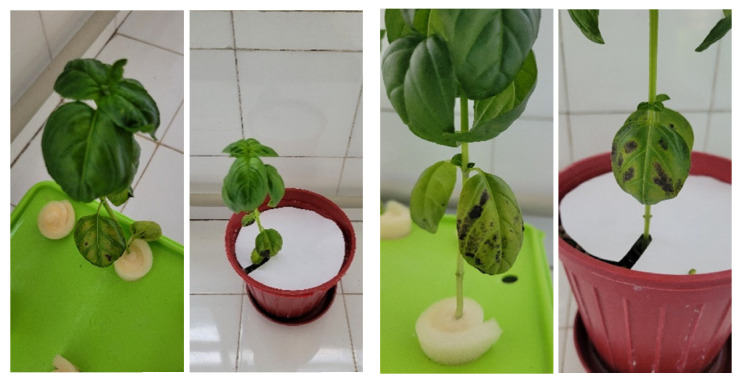
Plant-focused (**left**) and leaf-focused (**right**) images.

When symptoms appeared within 2 weeks after the experimental setup, photos were taken using a personal phone camera (Galaxy S20 FE SM-G780G, 12MP, F1.8) with an automatic setting of 3024 × 4032 pixels. Photographs were taken 3 days a week between 8.00 and 11.00 a.m. for plants showing symptoms of nutrient deficiency. Photographs (Figure 4) of a single plant were taken from different angles, leaf-focused and plant-focused, showing symptoms clearly. 

For each nutrient deficiency, 2 plants were separated for the test set and 4 plants were separated for the training set during the experiment. Additionally, this process was continued with the same groups throughout the experiment.

#### 2.2.2. Preparing Dataset

Since symptoms of calcium and magnesium deficiencies were unavailable, photos of these deficiencies (Ca- and Mg-) were not included in the dataset. The dataset consisted of 4 classes and 1757 photographs (Table 2) obtained from healthy plants and plants presenting nitrogen, phosphorus and potassium deficiencies. Symptom-based photographs are also included in the dataset. The main reason for the differences in the number of photographs between classes is that symptoms occur at different times in plants. The balance between the classes in the dataset was achieved in the preprocessing stage. 

The dataset was split into 2 groups, training and test, while the experiment was in progress. The number of images in the groups after the collection of the photographs is shown in Table 3. 

**Figure 5:**
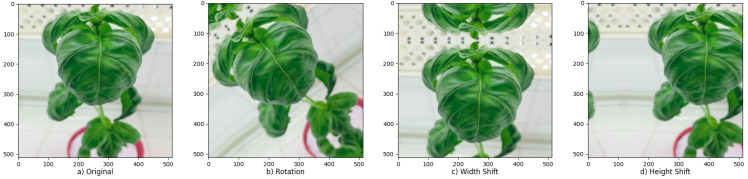

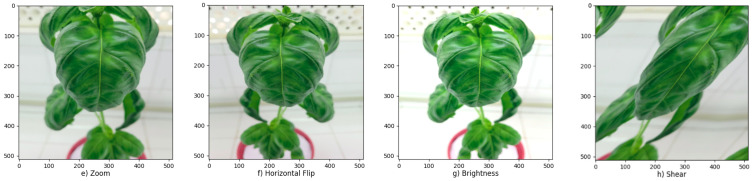
Applied data augmentation techniques.

Data augmentation (Figure 5) is a common CNN regularization technique used to prevent over-fitting. The number of samples and diversity in the dataset is increased with processes such as image rotation and reflection [25]. In this study, preprocessing for images was applied only to the images in the training set. Considering that color differences and the addition of noise in the photos would affect the symptoms, basic processes such as mirroring, shearing, rotation, zooming and cropping were applied to photos. In addition, the brightness value was changed in a short range (±0.1) during data augmentation due to the different levels of brightness in the original photos caused by weather conditions.

Before preprocessing, a total of 1125 images in the training set were duplicated to 10,000 images using the preprocessing methods mentioned above, the number of images has been seem in Table 4.

In order to prevent empty pixels that occur in photographs due to techniques such as shifting, zooming and shearing, the gaps were filled by taking the symmetry of the resulting image.

#### 2.2.3. Deep Learning and CNN

Depending on the data used, the depth added to artificial neural networks via deep learning not only adds complexity to the network but may also provide better learning ability, precision and high performance. Thanks to the large number of layers in this model, new features can be discovered and evaluated in each layer, and different features can be interpreted. This added depth enables the network to perform complex tasks successfully. In addition, deep learning makes it easy to work with big data, greatly automates feature extraction and saves it from interventions such as manually making sense of the dataset and choosing algorithms accordingly [26]. 

Convolutional neural networks are one of the most widely known and used types of deep learning. The CNN model is based on the imitation of human sight and the neural networks that form the visual cortex, which is why it has been commonly preferred in image and video processing research. The CNN model attempts to distinguish differences in multidimensional big data matrices received as input during training, then uses them in tasks such as object detection/segmentation, image classification, natural language processing, speech recognition and video processing [13,25]. The underlying properties of the CNN model, such as sparse interaction and weight sharing, make the CNN model useful for matrix inputs of two dimensions and above, such as image data. When the connection between intermediate layers is established between neurons that use the same features, this is called sparse interaction. Reducing the number of connections established reduces training speed and resource consumption. Weight sharing, on the other hand, is based on the logic that the parameters that are effective in extracting any given feature are shared with other neural cells, considering that extracted features of a certain region may be in a different location on a different image in cases where there is an input signal, such as an image. This process both reduces the number of parameters that need to be calculated and makes the model robust against sensitivities such as image shift, distortion and the presence of the feature in a different location [25].

Pre-trained neural network models are preferred in many CNN applications against problems such as the inadequacy of the dataset in terms of both quantity and diversity and the long training time of the CNN architecture designed for complicated problems [25,27]. The architecture and weights of the pre-trained model to perform a particular task are shared, and the feature extraction of this pre-trained model can be used to perform similar tasks. In this method, the main idea is the transfer of knowledge, called transfer learning [28]. DenseNet [14,17,29], AlexNet [14], VGG [14,17,19], ResNet [14,19,24], Inception-ResNet [15,17,30], Inception [19,30], EfficientNet [29,30] and MobileNet [29,30] are some of the learning transfer models that are commonly used in the literature and have been found by researchers to be successful in experiments related to plant health. Many different parameters, such as the depth and width of the models, the convolution layers used, the connections between the layers, etc., differentiate the architectures of the models and affect the performance of the models in factors such as success and training time. In many studies, the DenseNet architecture shows above-average performance [14,17,29,31], but the depth of the network and a large number of parameters increase the number of calculations and training time required during training. In this study, transfer learning models were preferred to test the new dataset because of their success in previous studies. In such cases, shallower models with fewer parameters, such as MobileNet [29,30,32], may be preferred depending on the task to be performed and the system on which the model will run.

#### 2.2.4. Grad-CAM

**Figure 6:**
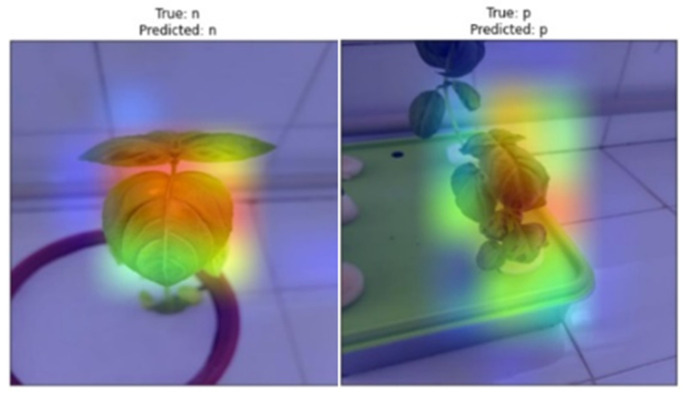
Heat maps obtained using Grad-CAM.

Grad-CAM is an algorithm that creates a heat map (Figure 6) to visualize which data and features a model focuses on the image input it uses for the task. It allows for the interpretation of the performance of the model by giving visual explanations of the image instead of the interpretation of the success of the model with the computational results obtained [33].

#### 2.2.5. Trained Details

In the study, the batch size was set to 64, and the input shape was set to (224, 224, 3). Before training of the model, preprocessing was applied exclusively to the images split for the training dataset, and new images were recorded. In addition, for applications with randomness, such as batch generation and dilution, the “seed” value was kept constant so that the models received the inputs similarly. “Imagenet” weights were set for DenseNet201, ResNet101V2, MobileNet and VGG16 pretrained CNN models. The epoch max value was selected as 100, but since the Early Stopping application was used in training, the training was terminated after 3 epochs depending on the changes in the “loss” value at different epoch values. Apart from the “loss” value, accuracy, precision and recall values were obtained during training. 

**Figure 7:**
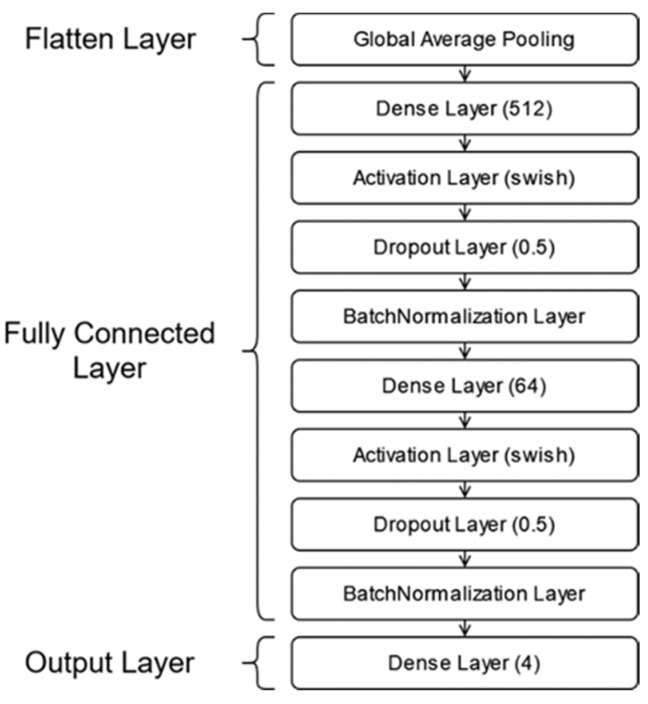
Designed, fully connected layer.

In the transition to the fully connected (FC) layer in the three models, flattening was performed with “GlobalAveragePooling”. In addition, the FC layer (Figure 7) contains 2 blocks consisting of “BatchNormalization”, “Dropout” and “Dense” layers. The “Swish” activation function was preferred for the “Dense” layers in the FC layer, while the “Softmax” activation function was preferred for the Dropout layer. The optimization function “Adam” and the loss function “categorical_crossentropy” were selected for training the model.

After the training, which was started by setting the epoch max value to 100, the models were fine-tuned by un-freezing the last layers of the models with a small learning rate of $1\times 10^{-5}$. The epoch max value was selected as 10 during the fine-tuning process, and the Early Stop application was set to monitor the “loss” value.

#### 2.2.6. Hardware and Software

This study was conducted with the Python programming language in the Jupyter Notebook development environment using a personal computer with an AMD Ryzen 7 4800H processor and NVIDIA GTX 1650Ti graphics card systems. Version 2.3.0 of the Tensorflow library was used in the deep learning applications and was set to allow for the use of the graphics card for computations.

## 3. Results and Discussion

In order for the models to be considered successful after training, their success in the given tasks was compared with quantitative performance measures such as confusion matrix (Table 5), accuracy (1), precision (2), sensitivity (3) and loss. The duration of training was also analyzed [20].

(1) $$Accuracy=\frac{\Sigma T+\Sigma T}{\Sigma T+\Sigma T+\Sigma F+\Sigma F}\times 100$$

(2) $$Precision=\frac{\Sigma T}{\Sigma T+\Sigma F}\times 100$$

(3) $$Recall=\frac{\Sigma T}{\Sigma T+\Sigma F}\times 100$$

**Figure 8:**
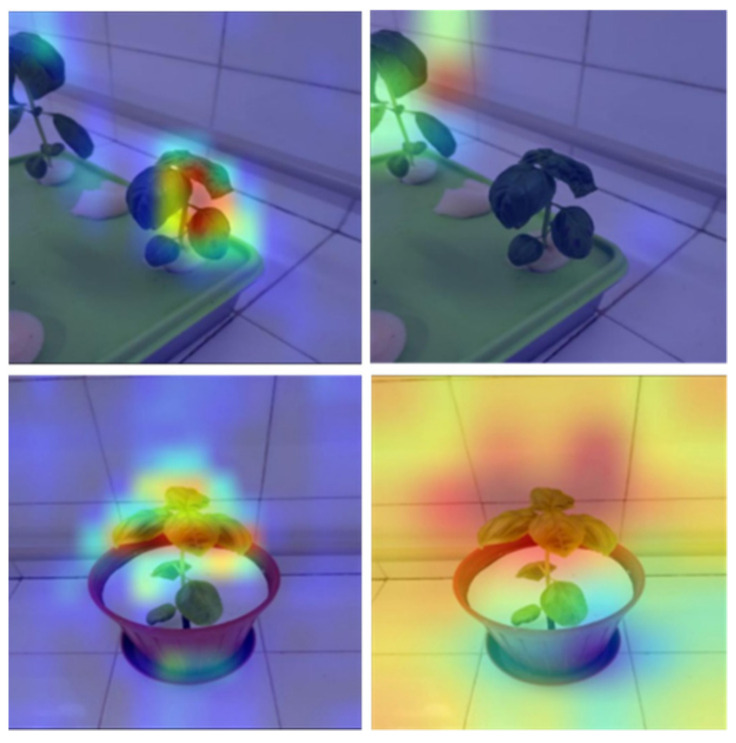
Successfully (**left**) and unsuccessfully (**right**) heat maps.

Heat maps (Figure 8) from the Grad-CAM approach were also evaluated as a qualitative performance measure. If the concentrations in the heat maps obtained from the models are on the plant/symptom, the model is assumed to have successfully extracted features.

Low loss and high accuracy are expected in the performance results obtained from the models. MobileNet completed the trainings (Table 6) faster than other models. A comparison of the loss and accuracy values of the models in the first training phase revealed that the MobileNet model achieved better results, with 96.86% accuracy and 0.090 loss. With fine-tuning, the VGG16 model achieved the lowest loss, 0.022, and the highest accuracy value, 99.89%. 

Except for VGG16, the performance of the models on the test set (Table 7) was close to their performance in the training phase (Table 6). The low performance values (Acc: 79.71%, loss: 0.601) obtained with VGG16 before fine-tuning gave the highest accuracy and the lowest loss values (Acc: 93.82%, loss: 0.185) among the models used in the study after fine-tuning. 

**Figure 9:**
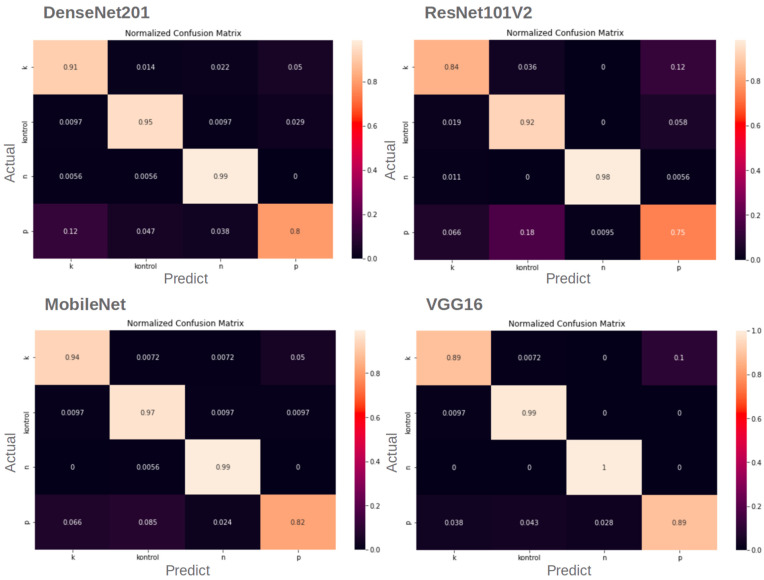
Confusion matrices of models.

Confusion matrices (Figure 9) show that the four CNN models make predictions for the N- and control classes with high accuracy, while the P- and K- classifications made by the models have lower accuracy levels. This is because the symptoms look like different class symptoms due to light differences in their images. This case was most common among P-, K- and control classes due to light and shadow conditions. 

**Figure 10:**
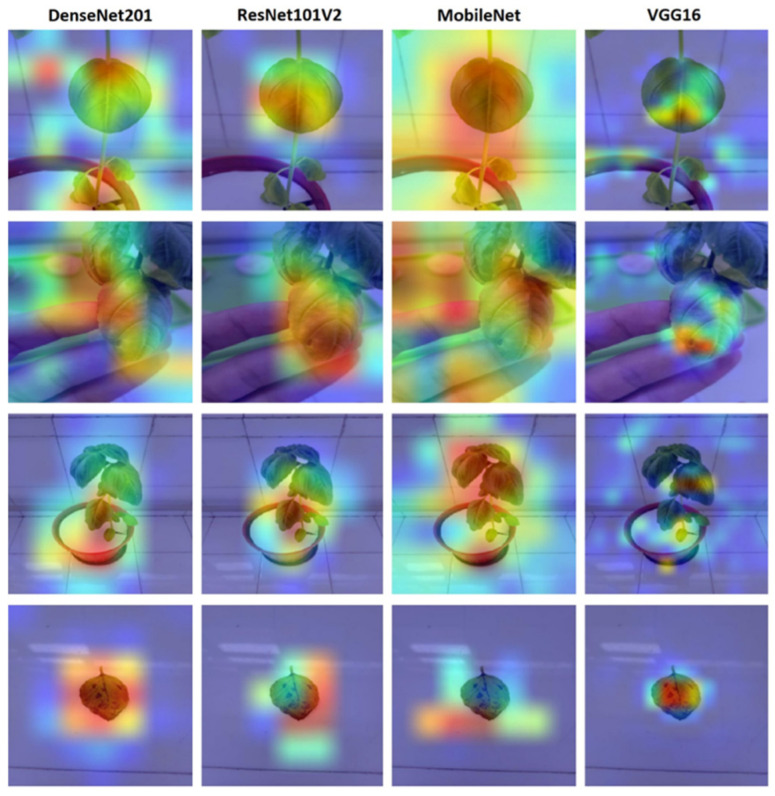
Heat maps of models.

The heat maps from Grad-CAM show which pixels on the image the models concentrate on to make their predictions. In heat maps, the models are expected to focus on the plants in the images and the symptoms of the plants. Although the MobileNet model performed close to the VGG16 model in terms of training time and accuracy, the pixels it used for classification mostly did not cover the plant. Analysis of the pixels selected by the VGG16, ResNet, and DenseNet models showed that these models were able to successfully identify the distinguishing features for classification for most images. As shown in Figure 10, analysis of the heat maps of the VGG16 model after fine-tuning revealed that it was able to focus on the symptoms.

The loss value must be as low as possible to ensure the reliability of the estimate of nutrient deficiency. Since “categorical crossentropy”, which we selected as the loss function, calculates the loss value depending on the probability of the classes, a lower loss value shows the reliability of the predictions and the probability of the models to make errors. In the test results (Table 7), Densenet201 has the lowest loss, 0.288, before fine-tuning. After fine-tuning, VGG16 has the most trustable predictions with 0.185 loss. 

We tested the dataset that we created in this study. Although the dataset contains different growth stages of basil plants, the models we used for the test that performed well in detecting nitrogen, phosphorus and potassium deficiencies in basil plants, demonstrating 89.54% accuracy and 0.288 loss. It can be seen that the results are even better with the fine-tuning process. Fine-tuning improves the results to 93.82% accuracy and reduces the loss to 0.185. After fine-tuning, the dataset gives more successful results in shallower CNNs. Analysis of the calculated loss values, accuracy (Table 7) and heat maps (Figure 10) reveals that VGG16 is the best model for our dataset considering the model’s performance after fine-tuning. When examining the complexity matrices, it is noticeable that adding more discriminative images for phosphorus and potassium deficiencies to the dataset could improve the results.

## 4. Conclusions

Incorrect fertilization in plant farming causes a decrease in product quality and yield and increases economic losses and pollution of the natural environment and resources. For correct fertilization, it is necessary to determine the deficient nutrients in the plant. The fact that nutrient deficiencies in plants manifest via physical symptoms allows us to use the CNN model to determine which element is deficient. Although the CNN model is successful in image processing, it is not widely used to control error-prone, real-time systems such as plantage, where misdirection can have negative consequences. A scarcity of data and lack of diversity are the main reasons for this. Therefore, we have created a new dataset of plant-oriented and leaf-oriented symptom images by setting up an experiment on nitrogen, phosphorus and potassium deficiencies in basil plants. The created dataset was tested with DenseNet201, ResNet101V2, MobileNet and VGG16 networks, and analysis was performed on the pixels that the models focused on when extracting features from images. In this study, difficulties were encountered, such as errors due to the presence of old-age symptoms in healthy plants in the dataset, the similarity of samples due to the early death of the plant with nitrogen deficiency and similarities between the symptoms of different element deficiencies due to light and shade. Despite this, the learning models proved successful in testing. Among the transfer learning models, DenseNet201 gave the best result, with 89.54% accuracy before fine-tuning, and analysis of the heat maps obtained from the Grad-CAM algorithm showed that it performed the classifications by focusing on the pixels covering the plant and symptoms in the images. The MobileNet model, which completed the training faster than the other models, showed high accuracy at a rate of 92.08% after fine-tuning, but analysis of the heat map showed that it used pixels that were not expected to be effective in classification. With the highest accuracy rate of 93.82% after fine-tuning, the VGG16 model focused on symptoms unlike the other models and achieved the highest performance for our dataset. In this study, we focused on testing a new plant to create a detailed dataset and we achieved successful results in our studies with transfer learning models. The basil plant grows within a short period of time, so it is helpful to add different growth stages to the dataset. In addition, the rapid response of the basil plant to changes in the environment speeds up the image acquisition process. These factors support the improvement and elaboration of the dataset by setting up new experiments on basil. In future studies, other nutrient deficiencies, especially Ca and Mg deficiencies, will be included in the dataset; when considering that more than one nutrient deficiency may occur at the same time, different combinations of deficiencies will be included in the dataset; symptoms of an excess of nutrients will be included in the dataset and the dataset will be diversified with these implementations. Then, a model will be trained to test in a greenhouse environment. Given the excess of some nutrients in the environment, an unsuitable pH value of the environment, etc., nutrient deficiencies, can be seen, so adding environmental and biotic causes to the system as additional data for detecting nutrient deficiency is planned. In addition, we planned to design a model that is superiorly successful in regard to nutrient deficiencies in basil and functions with the right pixels in feature extraction.

## Figures and Tables

**Table 1:** Recommend nutrient solution for aromatic plants.

**Element**	N	P	K	Ca	Mg	Fe	Mn	Zn	B	Cu	Mo
**Dosage (mg/L)**	180	50	210	180	50	4	0.5	0.1	0.5	0.1	0.05

**Table 2:** Number of photos belonging to classes.

Status of Plant	Number of Photos
Control	345
N-	456
P-	544
K-	412
Total	1757

**Table 3:** Number of photos in train set and test set.

	Number of Photos
Train Set	1126
Test Set	631

**Table 4:** Number of photos in unbalanced and balanced training sets.

		Training Set	
		Unbalanced Training Set	Balanced Training Set
Status of Plant	Control	242	2500
	N-	278	2500
	P-	333	2500
	K-	273	2500
	Total	1126	10,000

**Table 5:** Confusion matrix.

		Predict	
		Positive (Class_1)	Negative (Class_2)
Actual	Positive (Class _1)	True Positive (TP)	False Negative (FN)
	Negative (Class _2)	False Positive (FP)	True Negative (TN)

**Table 6:** The results of models from training phase.

	** Before Fine-Tuning **					
	**Loss**	**Acc** **(%)**	**Pre** **(%)**	**Rec** **(%)**	**Epoch**	**Time** **(s/epoch)**
DenseNet201	0.117	95.92	96.23	95.60	25	62
Resnet101V2	0.124	95.87	96.20	95.54	17	66
MobileNet	0.090	96.86	97.22	96.51	16	30
VGG16	0.293	89.11	90.42	87.91	34	53
	**After Fine-Tuning**					
DenseNet201	0.048	98.49	98.73	98.38	10	66
Resnet101V2	0.024	99.55	99.62	99.46	10	82
MobileNet	0.032	99.12	99.22	99.06	10	31
VGG16	0.022	99.89	99.91	99.88	10	81

**Table 7:** The results of models from test phase.

	** Before Fine-Tuning **			
	**Loss**	**Acc (%)**	**Pre (%)**	**Rec (%)**
DenseNet201	0.288	89.54	89.87	88.59
Resnet101V2	0.561	83.68	84.59	83.52
MobileNet	0.408	85.26	86.71	84.79
VGG16	0.601	79.71	82.06	78.29
	**After Fine-Tuning**			
DenseNet201	0.303	90.17	90.66	89.22
Resnet101V2	0.435	86.37	87.46	86.21
MobileNet	0.232	92.08	92.32	91.44
VGG16	0.185	93.82	94.36	92.87

## Data Availability

Not applicable.

## Abbreviations

AI	Artificial intelligence
Acc	Accuracy
Ca	Calcium
CNN	Convolutional neural network
K-	Potassium deficiency
K	Potassium
Mg	Magnesium
N-	Nitrogen deficiency
N	Nitrogen
P-	Phosphorous deficiency
P	Phosphorous
Pre	Precision
Rec	Recall
TP	True Positive
TN	True Negative
FP	False Positive
FN	False Negative

## References

[1] Kılavuz E., Erdem I. (2019). Dünyada tarim 4.0 uygulamalari ve Türk tariminin dönüşümü. Soc. Sci..

[2] Eryılmaz G.A., Kılıç O. (2018). Türkiye’de Sürdürülebilir Tarım ve İyi Tarım Uygulamaları. KSÜ Tarım Ve Doğa Derg..

[3] Kamyshova G., Osipov A., Gataullin S., Korchagin S., Ignar S., Gataullin T., Terekhova N., Suvorov S. (2022). Artificial Neural Networks and Computer Vision’s-Based Phytoindication Systems for Variable Rate Irrigation Improving. IEEE Access.

[4] Azimi S., Kaur T., Gandhi T.K. BAT Optimized CNN Model Identifies Water Stress in Chickpea Plant Shoot Images. Proceedings of the 2020 25th International Conference on Pattern Recognition (ICPR).

[5] Osipov A., Pleshakova E., Gataullin S., Korchagin S., Ivanov M., Finogeev A., Yadav V. (2022). Deep Learning Method for Recognition and Classification of Images from Video Recorders in Difficult Weather Conditions. Sustainability.

[6] Jung J., Maeda M., Chang A., Bhandari M., Ashapure A., Landivar-Bowles J. (2021). The potential of remote sensing and artificial intelligence as tools to improve the resilience of agriculture production systems. Curr. Opin. Biotechnol..

[7] Haq M.A. (2022). CNN Based Automated Weed Detection System Using UAV Imagery. Comput. Syst. Sci. Eng..

[8] Thenmozhi K., Reddy U.S. (2019). Crop pest classification based on deep convolutional neural network and transfer learning. Comput. Electron. Agric..

[9] Osipov A., Shumaev V., Ekielski A., Gataullin T., Suvorov S., Mishurov S., Gataullin S. (2022). Identification and Classification of Mechanical Damage During Continuous Harvesting of Root Crops Using Computer Vision Methods. IEEE Access.

[10] Korchagin S.A., Gataullin S.T., Osipov A.V., Smirnov M.V., Suvorov S.V., Serdechnyi D.V., Bublikov K.V. (2021). Development of an Optimal Algorithm for Detecting Damaged and Diseased Potato Tubers Moving along a Conveyor Belt Using Computer Vision Systems. Agronomy.

[11] Ayaşlıgil T.E., Çoşkun M.C. Sürdürülebilir Tarımda Topraksız Tarım ve Hidroponik Sistemlerin Önemi. Proceedings of the Akdeniz 7th International Congress on Applied Sciences.

[12] Gül A. (2019). Topraksız Yetiştiricilikte Bitki Besleme. Topraksız Tarım.

[13] Bhatt D., Patel C., Talsania H., Patel J., Vaghela R., Pandya S., Modi K., Ghayvat H. (2021). CNN Variants for Computer Vision: History, Architecture, Application, Challenges and Future Scope. Electronics.

[14] Yi J., Krusenbaum L., Unger P., Hüging H., Seidel S.J., Schaaf G., Gall J. (2020). Deep Learning for Non-Invasive Diagnosis of Nutrient Deficiencies in Sugar Beet Using RGB Images. Sensors.

[15] Wulandhari L.A., Gunawan A.A.S., Qurania A., Harsani P., Tarawan T.F., Hermawan R.F. (2019). Plant Nutrient Deficiency Detection Using Deep Convolutional Neural Network. ICIC Express Lett..

[16] Guerrero R., Renteros B., Castaneda R., Villanueva A., Belupu I. Detection of Nutrient Deficiencies in Banana Plants Using Deep Learning. Proceedings of the 2021 IEEE International Conference on Automation/XXIV Congress of the Chilean Association of Automatic Control (ICAACCA).

[17] Sharma M., Nath K., Sharma R.K., Kumar C.J., Chaudhary A. (2022). Ensemble Averaging of Transfer Learning Models for Identification of Nutritional Deficiency in Rice Plant. Electronics.

[18] Taha M.F., Abdalla A., ElMasry G., Gouda M., Zhou L., Zhao N., Liang N., Niu Z., Hassanein A., Al-Rejaie S. (2022). Using Deep Convolutional Neural Network for Image-Based Diagnosis of Nutrient Deficiencies in Plants Grown in Aquaponics. Chemosensors.

[19] Kusanur V., Chakravarthi V.S. (2021). Using Transfer Learning for Nutrient Deficiency Prediction and Classification in Tomato Plant. Int. J. Adv. Comput. Sci. Appl..

[20] Rahadiyan D., Hartati S., Wahyono, Nugroho A.P. (2022). Design of an Intelligent Hydroponics System to Identify Macronutrient Deficiencies in Chili. Int. J. Adv. Comput. Sci. Appl..

[21] Islam M., Hatou K., Aihara T., Seno S., Kirino S., Okamoto S. (2022). Performance prediction of tomato leaf disease by a series of parallel convolutional neural networks. SSRN Electron. J..

[22] Guo-Feng Y., Yong Y., Zi-Kang H., Xin-Yu Z., Yong H. (2022). A rapid, low-cost deep learning system to classify strawberry disease based on cloud service. J. Integr. Agric..

[23] Ngugi L.C., Abdelwahab M., Abo-Zahhad M. (2023). A new approach to learning and recognizing leaf diseases from individual lesions using convolutional neural networks. Inf. Process. Agric..

[24] Azimi S., Kaur T., Gandhi T.K. (2020). A deep learning approach to measure stress level in plants due to Nitrogen deficiency. Measurement.

[25] Alzubaidi L., Zhang J., Humaidi A.J., Al-Dujaili A., Duan Y., Al-Shamma O., Santamaría J., Fadhel M.A., Al-Amidie M., Farhan L. (2021). Review of deep learning: Concepts, CNN architectures, challenges, applications, future directions. J. Big Data.

[26] Shrestha A., Mahmood A. (2019). Review of Deep Learning Algorithms and Architectures. IEEE Access.

[27] Kamilaris A., Prenafeta-Boldú F.X. (2018). A review of the use of convolutional neural networks in agriculture. J. Agric. Sci..

[28] Zhuang F., Qi Z., Duan K., Xi D., Zhu Y., Zhu H., Xiong H., He Q. (2021). A Comprehensive Survey on Transfer Learning. Proc. IEEE.

[29] Espejo-Garcia B., Malounas I., Mylonas N., Kasimati A., Fountas S. (2022). Using EfficientNet and transfer learning for image-based diagnosis of nutrient deficiencies. Comput. Electron. Agric..

[30] Hassan S.M., Maji A.K., Jasiński M., Leonowicz Z., Jasińska E. (2021). Identification of Plant-Leaf Diseases Using CNN and Transfer-Learning Approach. Electronics.

[31] Huang G., Liu Z., Van Der Maaten L., Weinberger K.Q. Densely connected Convolutional Networks. Proceedings of the 2017 IEEE Conference on Computer Vision and Pattern Recognition (CVPR).

[32] Howard A.G., Zhu M., Chen B., Kalenichenko D., Wang W., Weyand T., Andreetto M., Adam H. (2017). MobileNets: Efficient convolutional neural networks for mobile vision applications. arXiv.

[33] Selvaraju R.R., Cogswell M., Das A., Vedantam R., Parikh D., Batra D. Grad-CAM: Visual Explanations from Deep Networks via Gradient-Based Localization. Proceedings of the 2017 IEEE International Conference on Computer Vision (ICCV).

